# Synapse alterations precede neuronal damage and storage pathology in a human cerebral organoid model of CLN3-juvenile neuronal ceroid lipofuscinosis

**DOI:** 10.1186/s40478-019-0871-7

**Published:** 2019-12-30

**Authors:** Gemma Gomez-Giro, Jonathan Arias-Fuenzalida, Javier Jarazo, Dagmar Zeuschner, Muhammad Ali, Nina Possemis, Silvia Bolognin, Rashi Halder, Christian Jäger, Willemijn F. E. Kuper, Peter M. van Hasselt, Holm Zaehres, Antonio del Sol, Herman van der Putten, Hans R. Schöler, Jens C. Schwamborn

**Affiliations:** 1 Luxembourg Centre for Systems Biomedicine (LCSB), Laboratory of Developmental and Cellular Biology, University of Luxembourg, Avenue du Swing 6, Belvaux, Luxembourg; 2 Laboratory of Cellular and Developmental Biologie, Max Planck Institute for Molecular Biomedicine, Röntgenstrasse, Münster, Germany; 3 Electron Microscopy Unit, Max Planck Institute for Molecular Biomedicine, Röntgenstrasse, Münster, Germany; 4 Luxembourg Centre for Systems Biomedicine (LCSB), Laboratory of Computational Biology, University of Luxembourg, Avenue du Swing 6, Belvaux, Luxembourg; 5 Department of Psychiatry and Neuropsychology, School for Mental Health and Neuroscience (MHeNS), Maastricht University, Maastricht, the Netherlands; 6 Luxembourg Centre for Systems Biomedicine (LCSB), Laboratory of Eco-Systems Biology, University of Luxembourg, Avenue des Hauts Fourneaux 7, Esch-sur-Alzette, Luxembourg; 7 Luxembourg Centre for Systems Biomedicine (LCSB), Laboratory of Enzymology and Metabolism, University of Luxembourg, Avenue du Swing 6, Esch-sur-Alzette, Luxembourg; 8 Department of Metabolic Diseases, Wilhelmina Children’s Hospital, University Medical Center Utrecht, Utrecht, the Netherlands; 9 Ruhr-University Bochum, Medical Faculty, Department of Anatomy and Molecular Embryology, Bochum, Germany; 10 IKERBASQUE, Basque Foundation for Science, Bilbao, Spain; 11 CIC bioGUNE, Bizkaia Technology Park, 801 Building, Derio, Spain; 12 NCL Foundation, Holstenwall 10, Hamburg, Germany

**Keywords:** JNCL, CLN3 disease, CRISPR/Cas9, Cerebral organoids, Neurodevelopment, Synapses

## Abstract

The juvenile form of neuronal ceroid Lipofuscinosis (JNCL) is the most common form within this group of rare lysosomal storage disorders, causing pediatric neurodegeneration. The genetic disorder, which is caused by recessive mutations affecting the *CLN3* gene, features progressive vision loss, cognitive and motor decline and other psychiatric conditions, seizure episodes, leading to premature death. Animal models have traditionally aid the understanding of the disease mechanisms and pathology and are very relevant for biomarker research and therapeutic testing. Nevertheless, there is a need for establishing reliable and predictive human cellular models to study the disease. Since patient material, particularly from children, is scarce and difficult to obtain, we generated an engineered a CLN3-mutant isogenic human induced pluripotent stem cell (hiPSC) line carrying the c.1054C → T pathologic variant, using state of the art CRISPR/Cas9 technology. To prove the suitability of the isogenic pair to model JNCL, we screened for disease-specific phenotypes in non-neuronal two-dimensional cell culture models as well as in cerebral brain organoids. Our data demonstrates that the sole introduction of the pathogenic variant gives rise to classical hallmarks of JNCL in vitro. Additionally, we discovered an alteration of the splicing caused by this particular mutation. Next, we derived cerebral organoids and used them as a neurodevelopmental model to study the particular effects of the CLN3^Q352X^ mutation during brain formation in the disease context. About half of the mutation -carrying cerebral organoids completely failed to develop normally. The other half, which escaped this severe defect were used for the analysis of more subtle alterations. In these escapers, whole-transcriptome analysis demonstrated early disease signatures, affecting pathways related to development, corticogenesis and synapses. Complementary metabolomics analysis confirmed decreased levels of cerebral tissue metabolites, some particularly relevant for synapse formation and neurotransmission, such as gamma-amino butyric acid (GABA). Our data suggests that a mutation in *CLN3* severely affects brain development. Furthermore, before disease onset, disease -associated neurodevelopmental changes, particular concerning synapse formation and function, occur.

## Introduction

Juvenile neuronal ceroid lipofuscinosis (JNCL), also commonly referred to as Batten disease or CLN3 disease (OMIM #204200) belongs to the neuronal ceroid lipofuscinoses: a larger group of lysosomal storage disorders which represent a major cause of neurodegeneration in children and young adults [6, 81]. With an estimated incidence range of 0.2–7 per 100,000 births worldwide, and an increased prevalence in northern European populations [38, 76], JNCL is considered a rare disease. Its symptoms typically manifest between 4 and 8 years of age with a rapid and progressive vision loss [59]. The disease advances with cognitive [42] and motor decline [43], accompanied by seizure episodes and behavioural changes, and inevitable leads to premature death during the second or the third decade of life, in the classical disease phenotype [71]. In addition to neurodegeneration, neurodevelopment may also be affected. Although animal models have broadened our knowledge about disease mechanisms, protein localization, function and interactions, the neurodevelopmental component to JNCL is still poorly understood. Only a few studies were able to show to some extent developmental abnormalities in newborn mice [58] or zebra fish embryos and larvae [78]. However, they might not recapitulate the features of the human disease.

JNCL is caused by recessively inherited mutations in the *CLN3* gene [33]. Up to date, a total number of 67 different mutations occurring in the *CLN3* gene have been compiled by the NCL Mutation and Patient Database (http://www.ucl.ac.uk/ncl/CLN3mutationtable.htm). Whereas most of the JNCL patients (80–85%) are homozygous for a 1.02 kb deletion of exons 7 and 8, compound heterozygous cases or homozygous for the different single nucleotide variants are scarce and usually manifest in one or few families [41]. The existence of CLN3 missense mutations that cause other disorders emphasises the need to study these variants closely [79]. Initial studies using patient-specific human induced pluripotent stem cells (hiPSCs) showing in vitro effects of *CLN3* mutations on the endocytic pathway and calcium homeostasis and autophagy have been published [18, 49]. However, patient-derived hiPSCs have the drawback that, aside from the disease-associated mutations, they carry the genetic background of the affected individuals, which can be extremely diverse between patients, making associating phenotypes directly to a particular gene mutation a complicated task. To overcome these limitations, we used state of the art CRISPR/Cas9 genome editing technologies [4] and introduced a disease-causing mutation into the *CLN3* gene of healthy hiPSCs. The newly generated isogenic pair represents an advantage compared to gene corrected cell lines [83], as it allows to study the contribution of a particular mutation to the disease phenotype, without any concomitant effect of the patient’s genetic background.

In this study, we used cerebral organoids as model for early brain development [45, 51] to investigate whether CLN3 deficiency affects fundamental neurodevelopmental mechanisms, such as growth and differentiation. Our results highlight transcriptional and metabolomic changes in CLN3 mutant organoids, when compared to controls, which indicate imbalances during brain development. Here, we provide a proof of principle that our cellular model recapitulates key disease features in different cell types in vitro and is thereby suitable for modeling JNCL.

## Results

### Generation of a CLN3 mutant isogenic pair

**Fig. 1:**
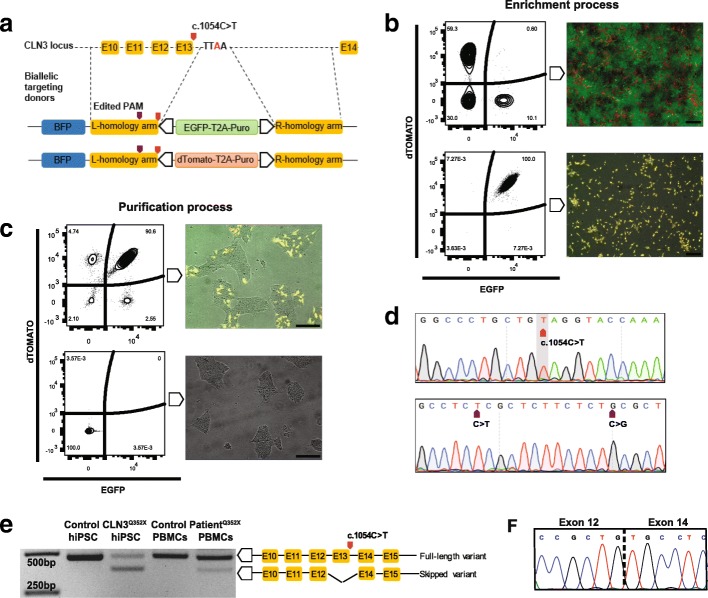
Gene editing-mediated introduction of the c.1054C > T mutation in the *CLN3* locus alters splicing. **a** Representation of biallelic donors containing distinctive fluorescent positive selection modules (PSM) and the targeted genomic region. **b** FACS plots depicting the different populations through the steps of the editing process. The upper panel represents the line after the integration of the PSM and the lower panel, the enrichment of the line after several rounds of sorting for the double-positive population. Plots are accompanied in the right side by microscope images for proper visualization of each step. Scale bars, 200 μm. **c** FACS plots depicting the different populations through the steps of the editing process. The upper panel represents the line after the excision of the PSM and the lower panel, the purification of the line after several rounds of sorting for the double-negative population. Plots are accompanied in the right side by microscope images for proper visualization of each step. Scale bars, 200 μm. **d** Sanger sequencing chromatogram of the obtained polyclone highlighting the introduction of the mutation and the silent PAM modifications. **e** Representative RT-PCR gel showing the different band amplification sizes in the CLN3^Q352X^ mutant hiPSCs and patient PBMCs, as compared to the controls. **f** Second band corresponds to an exon-skipping event of the mutated exon. Sanger sequencing confirmed the junction between neighboring exons

To insert the c.1054C → T pathologic variant on the *CLN3* gene, we designed a 21 bp sgRNA that targets the exon 13 of the human *CLN3* locus to produce a Cas9- induced double-strand break. In order to visualize and follow the genotypic outcome of the editing, excluding random integration, we applied the FACS assisted CRISPR-Cas9 genome editing (FACE) pipeline [3, 35]. Briefly, to promote homologous recombination, we created two double-stranded DNA donors containing a positive selection module with either EGFP or dTOMATO and the puromycin resistance gene, flanked by approximately 1 Kb homology arms. The left homology arm contained, in both donors, the c.1054C → T single nucleotide change for a homozygous outcome (Fig. 1a). In a first step, the constructs were introduced into the genome of healthy control hiPSCs. Puromycin-selected cells were collected, and a double-positive population was selected through several rounds of cell sorting, excluding the ones containing random integration events (BFP positive). Despite the fact that biallelic targeting occurred initially in a frequency of 0.6%, the entire population could be enriched (Fig. 1b). In a second step, the positive selection module was excised by exogenously expressing the excision-only variant of the PiggyBac transposase [47]. Subsequently, the double-negative population could be purified by cell sorting (Fig. 1c). Precise introduction of the mutation in homozygosis was confirmed by Sanger sequencing. Additionally, silent mutations in the PAM sequence, introduced to shield the site from Cas9 after insertion, were also present and in homozygosis, representing a successful editing procedure (Fig. 1d). Pluripotency of the lines was evaluated by immunostaining for OCT4, SOX2, TRA1–81, TRA1–60 and SSEA4 (Additional file 1: Figure S1b). Karyotype assessment revealed no major abnormalities in both the edited polyclone and the parental control lines (Additional file 1: Figure S1c). Potential off-target loci for the utilized sgRNA (refer to CLN3-QtoO-B in methods section) were predicted by the CRISPR/Cas9 target online predictor, CCTOP [73]. For the top seven off-target sites, we designed a pair of primers to amplify between 200 and 900 bp of the predicted region. Sanger sequencing analysis revealed no detectable off-target modifications induced in the isogenic pair by the genome editing process (Additional file 6: Table S2).

### The *CLN3* p.Q352X mutation causes a novel splicing variant

The c.1054C → T mutation is located at the last codon of exon 13 from the *CLN3* gene and it produces a premature termination codon (PTC), responsible for generating a truncated protein [55]. Brooks and colleagues reported a frequency of 2.8% of PTC-inducing mutations for the *CLN3* gene [12]. In addition, PTCs are shown to frequently induce alternative splicing, often leading to skipping of the PTC-containing exon [17]. To test this hypothesis in the here generated isogenic line, reverse-transcription PCR (RT-PCR) analysis using primers that amplified exons 11 to 15, revealed two different cDNA amplicons in the *CLN3* mutant hiPSCs, one with the expected normal size (480 bp) and a product of around 100 bp smaller in size (Fig. 1e). Sanger sequencing confirmed that the smaller product corresponded to an exon-skipped variant, which lacked exon 13 (Fig. 1f). To our knowledge, the potential affection of the splicing by the p.Q352X mutation was not previously described. Therefore, we further investigated this alternative splicing variant in peripheral blood mononuclear cells (PBMCs) extracted from a patient carrying the same mutation in homozygosity. This confirmed the same splicing pattern, although the proportion of the two variants seems to differ, which might be caused by cell type specific differences (Fig. 1e).

### CLN3^Q352X^ hiPSC-derived endothelial cells recapitulate JNCL disease hallmarks

**Fig. 2:**
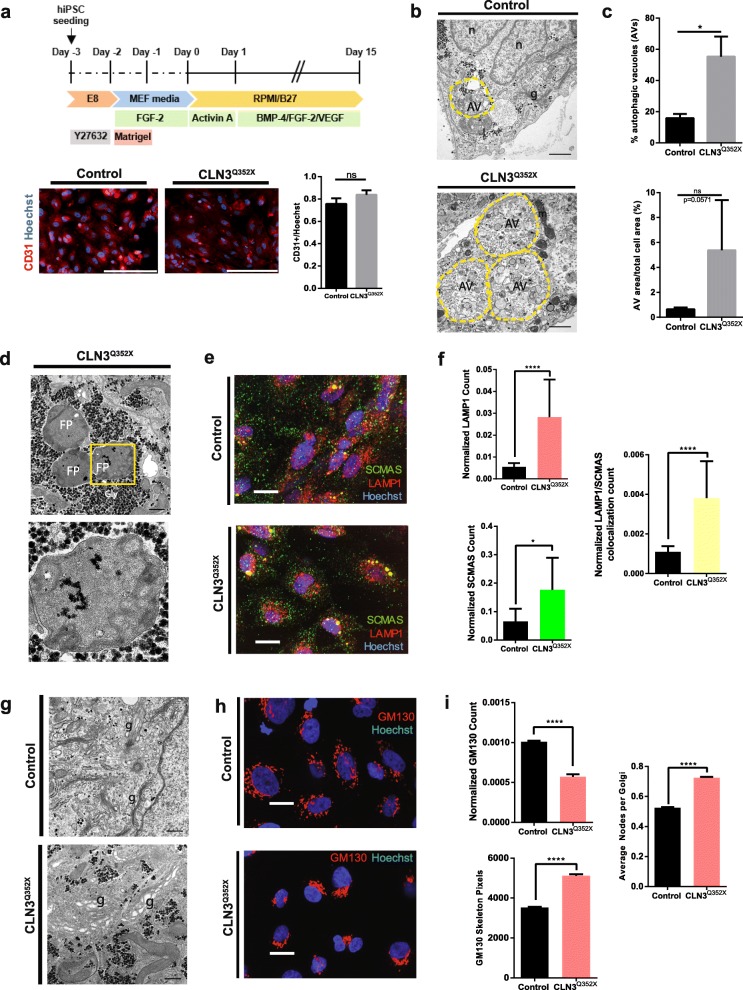
The *CLN3* c.1054C > T introduction generated classical JNCL phenotypes in vitro. **a** Protocol for endothelial cell (EC) derivation from hiPSCs and representative immunofluorescence pictures of CD31 positive endothelial cells for characterization. Scale bar, 200 μm. Quantification of CD31 positive cells performed in Control and CLN3^Q352X^ ECs. Mean fluorescence intensity of the CD31 channel normalized to the nuclear (Hoechst positive) signal was calculated for 12 random fields per condition using ImageJ (NIH) (Welch’s test; ns, not significant). Data is shown as mean ± SEM. **b** Representative TEM pictures highlighting increased presence of autophagic vacuoles (AVs, yellow dashed line) in the CLN3^Q352X^ ECs. Other organelles (n, nuclei; g, Golgi; l, lysosome; m, mitochondria). Scale bars, 1 μm. **b** Quantification of the percentage of AVs evaluated as the number of events in 56 Control and 52 CLN3^Q352X^ random cell profiles. Area of the AVs was calculated on ImageJ (NIH) by manually delineating the perimeter as regions of interest (ROI). AV areas were normalized to the total area of the cell containing them. All measurements were corrected for the magnification of the pictures if needed. Comparison between genotypes was performed by a Mann-Whitney test (*p < 0.05; ns; not significant). Data is shown as mean ± SEM. **d** Representative TEM pictures of fingerprint inclusions (FPPs) seen in CLN3^Q352X^ ECs (Gly, glycogen deposits). Scale bar, 500 nm. Zoomed in picture (yellow dashed square) for better visualization of membrane stacks. Scale bar, 200 nm. **e** High-content imaging of SCMAS (green), LAMP1 (red) structures, and co-localization events (yellow) in Control and CLN3^Q352X^ ECs. Scale bar, 200 μm. **f** SCMAS, LAMP1 and colocalization counts normalized to the nuclear (Hoechst positive) area of each field. Differences were evaluated using Mann-Whitney test (*p < 0.05, ****p < 0.0001). Data is represented as mean ± SEM of three technical replicates. **g** Representative TEM images of structural differences in the Golgi compartment in the Control (stacked) and the CLN3^Q352X^ (dilated) ECs (g, Golgi; m, mitochondria; gly, glycogen). Scale bars, 500 nm. **h** High-content imaging of Golgi (GM130 positive) structures (red). Scale bar, 200 μm. **i** Quantification of GM130 counts, normalized to the nuclear (Hoechst positive) area of each field. GM130 positive structures present an increased ramification in terms of skeleton pixels and average nodes per Golgi structure. Comparison performed with Mann-Whitney test (****p < 0.0001). Data is represented as mean ± SEM of three technical replicates

In juvenile neuronal ceroid lipofuscinosis the ultrastructural visualization of storage material, in a distinctive pattern, called fingerprint profiles (FPPs) constitutes a disease hallmark. Following the study from Pérez-Poyato and colleagues, that used endothelial cells (ECs) to characterize storage inclusions in CLN3 patients [62], we derived endothelial cells (ECs) from the here generated hiPSC isogenic pair and investigated the potency of the CLN3^Q352X^ mutant line to recapitulate storage disorder pathology. Cell differentiation towards the endothelial lineage was assessed by immunostaining of the endothelial cell surface marker CD31, showing a similar staining distribution in both cell lines (with a mean fluorescence intensity of 0.76 ± 0.05 in the Control and 0.84 ± 0.04 in the CLN3^Q352X^ ECs) (Fig. 2a). The different cellular compartments and organelles were examined by transmission electron microscopy (TEM) after 15 days of differentiation. We observed major autophagic structures, constituted by a single limiting membrane and cellular components at various stages of degradation, which represent autophagic vacuoles (AVs) (Fig. 2b). Quantification of the percentage of AVs per cell profile highlighted a significantly increased presence of these structures in the CLN3^Q352X^ mutant line (15.57 ± 2.7% in the Control and 55.27 ± 12.9% in the CLN3^Q352X^ ECs). Although the AV area normalized to total cell area was not significantly different, there was a trend towards an increased size of the AVs in the case of the mutant cells (0.64 ± 0.15% in the Control versus 5.38 ± 4.02% in the CLN3^Q352X^ ECs) (Fig. 2c). Strikingly, we were also able to find electron dense storage material with fingerprint patterned morphology in the CLN3^Q352X^ mutant ECs, which looked very similar to the storage material typically observed in JNCL patients (Fig. 2d) [49, 54]. The observation of the classical storage material by TEM prompted us to further examine the ECs by immunostaining of the subunit c of the mitochondrial ATP synthase (SCMAS) and the lysosomal-associated membrane protein 1 (LAMP1). We observed a significantly higher amount of LAMP1 and SCMAS positive structures in the CLN3^Q352X^ mutant ECs, as well as a significantly higher degree of co-localization between the two structures, indicative of an increased presence of SCMAS inside the lysosomes in the case of the mutant ECs, which may be part of the aberrant storage material (Fig. 2e, f). Alterations were not only restricted to the lysosomal compartment. Golgi stacks were often broadened up in the case of the CLN3^Q352X^ mutant cells (Fig. 2g). Consequently, we immunostained to visualize the *cis*-Golgi marker GM130 (Fig. 2h). Image analysis revealed a lower proportion of Golgi structures in the CLN3^Q352X^ mutant ECs but with a higher degree of ramification, as seen by the increased amount of pixels that form the Golgi skeleton and the average of ramification points (or nodes) per Golgi structure (Fig. 2i). Evaluation of other organelles revealed no ultrastructural abnormalities in the endoplasmic reticulum (ER) (Additional file 2: Figure S2a), the endosomal membranes (early endosomes and late endosomes/lysosomes) (Additional file 2: Figure S2b), or the mitochondria (Additional file 2: Figure S2c) in the CLN3^Q352X^ ECs. However, immunostaining for the Ras-related protein Rab-7a (RAB7) (Additional file 2: Figure S2d), a small GTPase that plays a key role in regulating the transport from early to late endosomal compartments [29], highlighted a significantly decreased amount of RAB7 positive structures, which are also smaller in size on average, in the CLN3^Q352X^ ECs compared to the Control cells (Additional file 1: Figure S2e). As RAB7 is a key regulator of the transport into degradative compartments [15], these results are a further indication for defects in the maturation and/or trafficking of the endo-lysosomal compartments in CLN3^Q352X^ ECs.

### Severe failure to develop in CLN3^Q352X^ hiPSC-derived cerebral organoids

**Fig. 3:**
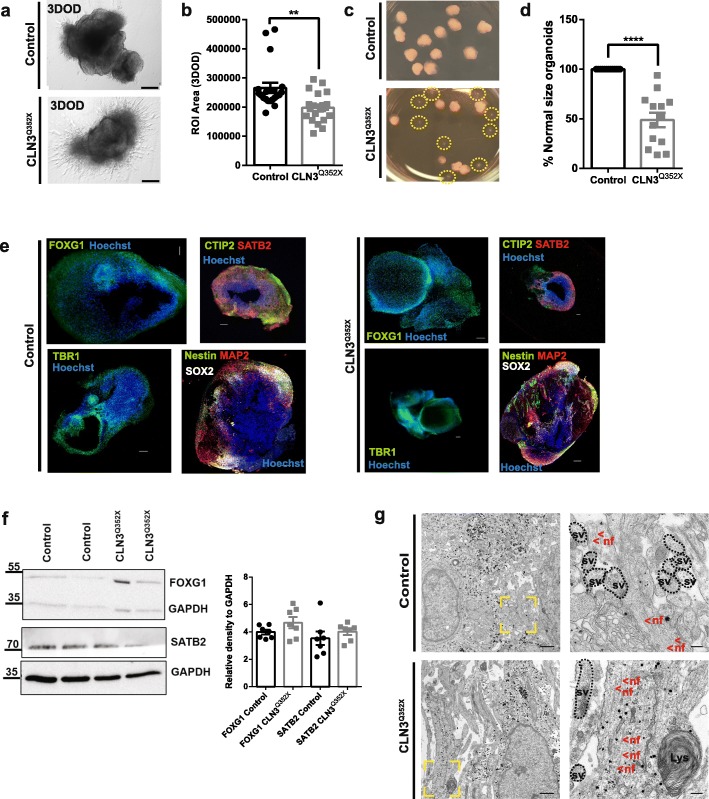
A large fraction of CLN3^Q352X^ hiPSC-derived cerebral organoids exhibit impaired growth. **a** CLN3^Q352X^ organoids fail to expand and form neuroectodermal protrusions at three days of differentiation (3DOD) compared to the Control organoids. Scale bar, 200 μm. **b** Size measurements of organoids at 3DOD show a decrease in the area of the CLN3^Q352X^ organoids. Area was calculated on ImageJ (NIH) by manually delineating the perimeter as regions of interest (ROI). Data points represent single organoids from three independent derivations (total *n* = 20 organoids). Comparison was performed applying a Mann-Whitney test between the two groups. **c** A proportion of CLN3^Q352X^ organoids remains underdeveloped after 55DOD. Small Matrigel-embeded organoids are highlighted with a dashed yellow circle. **d** Quantification of the percentage of CLN3^Q352X^ mutant organoids reaching a complete development. Data points represent percentage per organoid derivation (total *n* = 13 different organoid generations). Comparison between the two groups was performed with an unpaired t-test with Welch’s correction. For B and D, data is represented as mean ± SEM (*p* < 0.01**, *p* < 0.0001****). **e** Fully developed organoids from both Control and CLN3^Q352X^ mutant genotypes express forebrain marker FOXG1 (green) and cortical layer identities: SATB2 (red) for later-born superficial-layer identity and CTIP2 and TBR1 for early-born deep-layer identity (CTIP2 and TBR1, both green), at 55DOD. They also express the neuronal marker MAP 2 (red) and neural stem cell markers SOX2 (white) and Nestin (green). Scale bars, 200 μm. **f**. Representative western blots for the some of the markers mentioned above. Quantifications show similar expression in fully developed CLN3^Q352X^ organoids compared to the Controls at 55DOD. Data points represent single different organoids (*n* = 7 different organoids per condition). **g** Representative TEM pictures of the ultrastructural neuronal features of both organoid cultures at the same stage. Nerve fibers are highlighted in a yellow dashed boxed and higher magnification picture is offered at the right side to visualize neurofilaments (nf; <, both in red), which are present in both genotypes, as well as synaptic vesicles (sv; black dashed circles). Scale bars represent 1 μm, 200 nm for magnified areas

Little is known about early brain development in the context of JNCL. To evaluate whether there is an early developmental component to CLN3 disease, we used cerebral organoids as a state of the art human model to study brain development and disease [1, 45, 51, 66]. In this regard, we generated cerebral organoids from the here generated hiPSC isogenic pair [44] and we cultured them up to the stage where cortical markers are typically expressed (55 days of differentiation) [69]. Initial screening involving size evaluation of individual organoids up to the stage of neural induction (day 10) revealed no major differences (Additional file 3: Figures S3a, b). However, upon matrix embedding and initiation of the differentiation (day 11), the average size of CLN3^Q352X^ organoids was slightly, but significantly reduced, in comparison to the isogenic control without the mutation (Fig. 3a, b). Interestingly, about half of the CLN3^Q352X^ organoids completely failed to grow and develop further until the end-point of the differentiation (Fig. 3c, d). This severe phenotype was observed repeatedly across several organoid generations. The other half of mutant organoids, which escaped this severe defect were used for the analysis of more subtle alterations. In these fully-developed cerebral organoids, with and without mutant CLN3, comparable expression of brain identity markers, such as the forebrain marker FOXG1 were detected, either via immunofluorescence or western blot. Neuronal marker MAP 2 was also present and neuronal identity corresponding to several cortical layers, like SATB2 expression for later-born superficial-layer identity neurons, and early-born deep-layer identity marked by the expression of CTIP2 and TBR1 were detectable at comparable levels. Moreover, they also expressed neural stem cell markers SOX2 and Nestin (Fig. 3e, f) [68]. However, mRNA levels of *FOXG1*, *SATB2* and *TBR1* genes were found significantly downregulated in the CLN3^Q352X^ mutant phenotype (Additional file 5: Figure S5a). Finally, TEM analysis highlighted the presence of nerve fibers with neurofilaments and synaptic vesicles in both genotypes (Fig. 3g). Based on these results we conclude that about half of the CLN3^Q352X^ organoids were able to escape severe development defects which prohibit their development into cerebral organoids. We decided to further analyse these escapers for more subtle defects that might be caused by presence of the CLN3^Q352X^ mutation.

### CLN3 mutant cerebral organoids present lysosomal alterations, storage pathology and astrocytosis

**Fig. 4:**
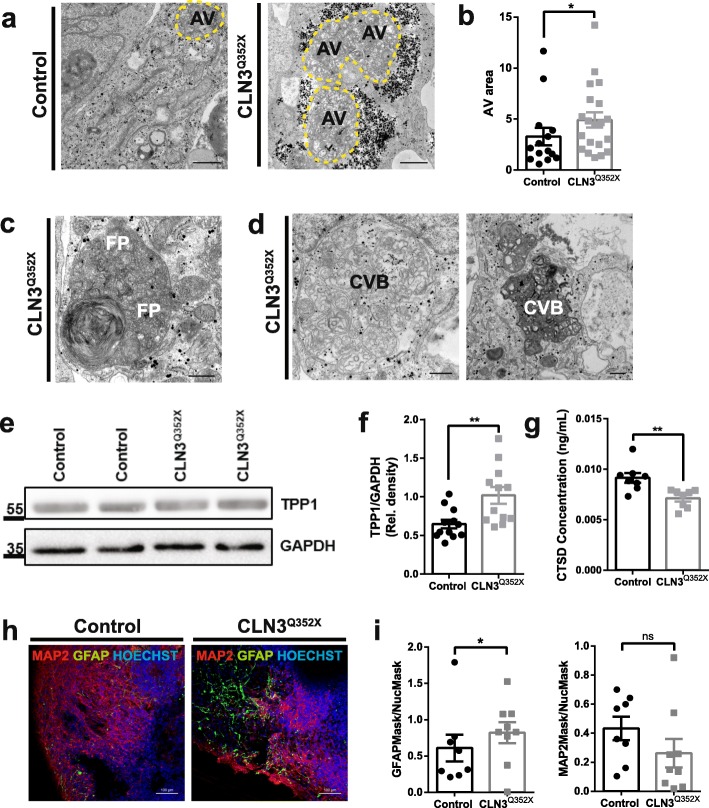
JNCL cerebral organoids recapitulate disease hallmarks in the neuronal tissue. **a** Representative TEM pictures highlighting increased presence of autophagic vacuoles (AVs, dashed yellow line) in the CLN3^Q352X^ organoids. Scale bars, 1 μm. **b** Quantification of AV area, measured on ImageJ by region of interest (ROI) definition, reveals a significant increase in the size of the vacuoles in the CLN3^Q352X^ organoids. Comparison from 14 Control and 20 CLN3^Q352X^ random AV profiles was done using Mann-Whitney test, *p < 0.05. Data points indicate single AVs measured per condition. **c** Representative TEM pictures of fingerprint inclusions (FPPs) detected exclusively in the CLN3^Q352X^ organoids. Scale bar, 500 nm. **d** Representative EM pictures of curvilinear bodies (CVB) found exclusively in the CLN3^Q352X^ organoids. Scale bar, 500 nm. **e** Representative western blot staining for TPP1 showing a slight increase in the CLN3^Q352X^ organoids. **f** Quantification of the TPP1 levels from western blots. Data points represent individual organoids (*n* = 12 organoids from at least three independent derivations) (Welch’s test; **p < 0.01). **g** ELISA quantification of CTSD protein levels. Data points represent single different organoids (*n* = 8 organoids generated in at least three independent derivations) (Welch’s test; **p < 0.01). **h** Astrocytosis shown by increased GFAP positive (green) staining in the CLN3^Q352X^ organoids compared to the controls. Scale bars, 100 μm. **i** Image analysis of GFAP and MAP 2 positive staining in organoid sections, normalized by nuclear (Hoechst positive) staining. Data points represent different organoid sections (*n* = 8 Control and 9 CLN3^Q352X^ sections taken from different organoids generated in at least three independent derivations). Comparison evaluated by Kolmogorov-Smirnov test (*p < 0.05; ns, not significant). Data in B, F, G and I is shown as mean ± SEM

Ultrastructural analysis of CLN3^Q352X^ mutant cerebral organoids confirmed the presence of pathological storage material, as seen by the increased presence of AVs (Fig. 4a) of significantly greater area in the case of the CLN3^Q352X^ organoids (4.9 ± 0.75) compared to the Control organoids (3.28 ± 0.86) (Fig. 4b). Strikingly, we were also able to find intracytoplasmic and electron dense storage material with fingerprint morphology in the CLN3^Q352X^ organoids mutant organoids [54], as well as structures resembling curvilinear bodies (CVB) [9] (Fig. 4c, d). Pathological intracellular deposits in JNCL are typically autofluorescent [70] and composed mainly of subunit c of the mitochondrial ATPase (SCMAS) [60]. However, autofluorescence analysis in organoid sections with confocal laser excitation at 568 nm [52] did not reveal any increase in the CLN3^Q352X^ mutant organoids (Additional file 3: Figures S3c, d). SCMAS protein levels were also not significantly increased (Additional file 3: Figures S3e, f). To determine whether the storage material results in an impairment of lysosomal functionality, we analyzed the protein levels of TPP1/CLN2, a serine protease in the lysosome, which interacts with CLN3 [77], and found a significant increase in CLN3^Q352X^ organoids (Fig. 4e, f). However, at the gene expression level, mRNA counts for the *TPP1* gene were significantly decreased in the CLN3^Q352X^ mutant organoids (Additional file 5: Figure S5b). Additionally, CLN3 has also been implicated in the regulation of other proteases in the lysosome, including Cathepsin D (CTSD/CLN10) [16, 26]. We detected a significantly decreased concentration of CTSD in lysates from CLN3^Q352X^ cerebral organoids (Fig. 4g), suggesting that functional alterations at the lysosomal level are already present at this time-point in this developmental model. This is important because not only these proteins are thought to interact with the *CLN3* gene, but mutations in TPP1, as well as CTSD cause late-infantile and congenital NCL, respectively [6]. Additionally, we found increased astrocytosis, indicated by the significant increase of GFAP+ cells, in CLN3^Q352X^ mutant organoids (Fig. 4h, i), which has been often reported in *CLN3* deficient mice brains [8, 61, 63]. Differences in MAP 2-positive neuronal areas were not significant, however, a trend towards a decreased neuronal areas that may be due to an increased astrocytosis could be observed (Fig. 4i). Furthermore, we were not able to detect increased percentages of apoptotic TUNEL+ DAPI+ cells in CLN3^Q352X^ organoids (Additional file 3: Figures S3 g and h), and programmed necrosis (or necroptosis) marker levels, such as kinases receptor-interacting proteins 1 and 3 (RIP1 and RIP3) [7], were not significantly different from the ones in the Control organoids (Additional file 3: Figures S3i, j). In summary, these data reveal that we can detect disease relevant early alterations, particularly concerning lysosomal function in CLN3^Q352X^ mutant cerebral organoids, which may precede more severe phenotypes including cell death.

### Whole-transcriptome analysis reveals impaired development in CLN3^Q352X^ cerebral organoids

**Fig. 5:**
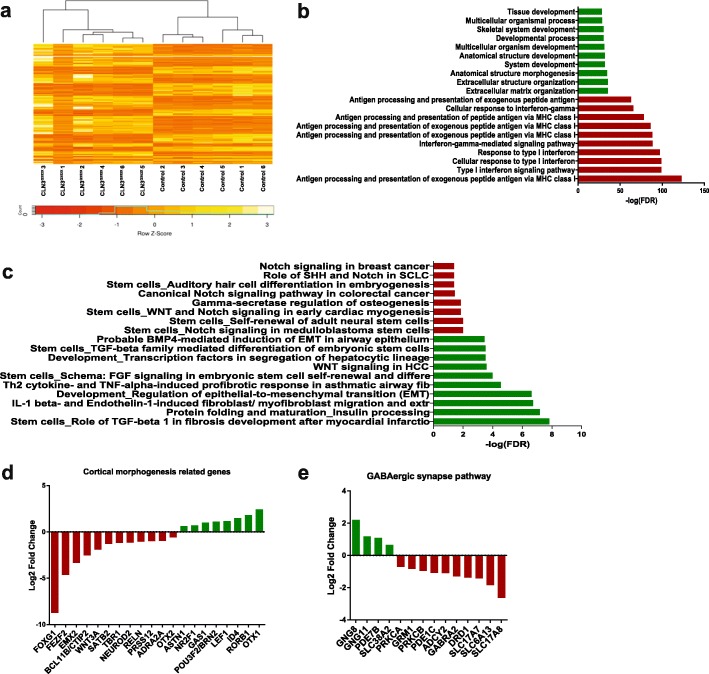
Whole-transcriptome analysis reveals impaired development in CLN3^Q352X^ cerebral organoids. **a** Heatmap clustering the differentially expressed genes between Control (healthy) and CLN3^Q352X^ (mutant) brain organoids. **b** Gene enrichment analysis of CLN3^Q352X^ network. Genes that are upregulated in disease phenotype were significantly enriched in cellular processes highlighted in green, while downregulations are depicted in red. **c** Pathway enrichment analysis of the CLN3^Q352X^ network. Pathway up-regulations are highlighted in green, while down-regulations are marked in red. **d** Log 2 Fold change expression values for genes related to brain development and cortical morphogenesis were generally decreased in CLN3^Q352X^. **e** Log 2 Fold change expression values for genes related to synapses, which were mostly downregulated in the CLN3^Q352X^ organoids. Genes in D and E are specifically differentially in our dataset (but not necessarily present in the networks) and belong to the pathways extracted from Pathway unification database (PathCards)

Comparative transcriptomic analyses in the context of CLN3 disease are very limited [32]. Therefore, we used those CLN3 mutant organoids that were able to develop and performed whole-transcriptome RNA-seq analysis to detect early disease signatures at the gene expression level. In order to gain a deeper understanding of JNCL disease-related dysregulations at a systems-level, we employed a differential gene regulatory network (GRN) based analysis to reconstruct phenotype-specific networks representing the CLN3^Q352X^-diseased (mutant) and Control (healthy) phenotypes [5, 14, 65]. Differential expression analysis (DEA) resulted in 972 genes to be significantly (Benjamini Hochberg corrected *p*-value ≤0.05 and logFC > 1) differentially expressed (up- and down-regulated) between the Control and the CLN3^Q352X^ mutant cerebral organoids (Fig. 5a). The hierarchical clustering analysis revealed the presence of two clearly distinct groups, with different gene expression signatures, which correlate with the presence of the CLN3^Q352X^ mutation (Fig. 5a). The reconstructed CLN3^Q352X^-diseased network comprised 353 genes and 641 interactions, whereas the Control healthy network contained 298 genes and 399 interactions (Additional file 4: Figures S4a, b). Interestingly, gene ontology (GO) analysis of the CLN3^Q352X^–diseased network revealed that most of the up-regulated genes in the network were significantly enriched in cellular processes related to development, such as tissue development (GO:0009888, FDR:5.84E-29), multicellular organism development (GO:0007275, FDR:6.67E-32) and extracellular matrix (ECM) organization (GO:0030198, FDR:3.68E-36). On the other hand, most downregulations were targeting biological processes related to antigen processing and presentation via the class I major histocompatibility complex (MHC I) (GO:0002480), FDR: 9.73E-124) (Fig. 5b). Interestingly, antigen presenting cell abnormalities have been previously reported in a CLN3-deficient mouse model [31]. Furthermore, pathway enrichment analysis highlighted significant dysregulations in molecular pathways related to stem cells and development (Fig. 5c). Particularly, the TGF-beta, WNT and BMP signaling pathways were found to be significantly associated with the disease specific network, these signaling pathways are well characterized for their fundamental roles in embryonic development and homeostasis [72, 80, 82]. This suggested that dysregulations of developmental pathways and processes in CLN3^Q352X^ cerebral organoids constitute a disease signature, already at early stages during the brain development. Additionally, we analyzed the expression levels of genes related to cortical morphogenesis and found that transcription factors critical in central nervous system development, such as FOXG1 [30], FEFZ2 [22], CTIP2 [56]*,* SATB2 [11], TBR1 [21] or NEUROD2 [57] were predominantly downregulated in the CLN3^Q352X^ organoids (Fig. 5d). Moreover, expression of genes encoding relevant synaptic proteins, such as amino acid transporters from the SLC6 and the SLC17 families, as well as neurotransmitter receptors, such as γ-Aminobutyric acid (GABA) receptor GABRA2 and dopamine receptor DRD1, were also decreased (Fig. 5e). These downregulations are indicative of specific alterations in cortical neuronal specification and synapse formation in CLN3^Q352X^ organoids.

### CLN3^Q352X^ cerebral organoids display early synaptic and neurotransmitter defects

**Fig. 6:**
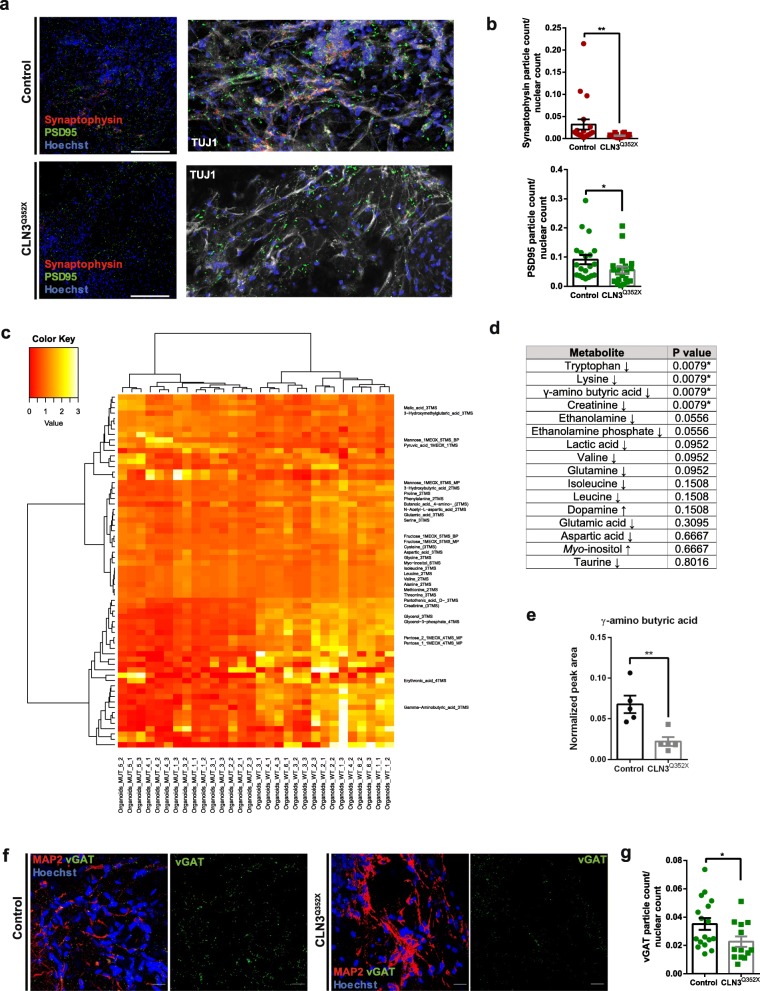
Dysregulations at the synapses might precede other phenotypes in CLN3^Q352X^ cerebral organoids. **a** Representative confocal images of synaptic protein staining for Synaptophysin (red) and PSD95 (green). Scale bars, 200 μm. Zoomed regions with TUJ1 (white) neuronal staining. **b** Quantification of the Synaptophysin and PSD95 positive particles normalized to nuclear (Hoechst positive) count. Two regions of interest (ROI) per organoid section were imaged. Following automatic thresholding, the particle analyzer tool from ImageJ (NIH) was used to quantify punctate stainings -PSD95 and Synaptophysin- and the ITCN nuclear count tool to quantify Hoechst positive nuclei. Each data point represents a region of interest (ROI). Total n per group equals 10 sections taken from organoids generated in at least three independent derivations. Comparison between groups was performed with a Mann-Whitney test (**p < 0.01; ns, not significant). **c** Heatmap representing hierarchical clustering of deregulated metabolites between the Control and the CLN3^Q352X^ mutant organoids. 5 different pools of 3 organoids were analyzed and 3 technical replicates per measurement were performed. **d** Table containing cerebral tissue metabolites. Arrows indicate relative increase or decrease in the CLN3^Q352X^ mutant organoids compared to the controls. Asterisks mark significantly deregulated metabolites in the mutant organoids, corresponding to *p* values. **e** Neurotransmitter GABA is particularly downregulated in the CLN3^Q352X^ mutant organoids. Significant differences were evaluated with a Mann-Whitney test comparison (**p < 0.01). Data points represent the average of technical replicates per organoid pool. Data in B and E is shown as mean ± SEM. **f** Representative confocal images of inhibitory GABAergic neurons pre-synaptic protein vGAT staining (green) and MAP 2 positive neuronal areas (red). Single vGAT channel is displayed inside a square dashed box for better visualization. Scale bars, 20 μm. **g** Quantification of the vGAT positive particles normalized to Hoechst positive nuclei. Several regions of interest (ROI) per organoid section were imaged. Following automatic thresholding, the particle analyzer tool from ImageJ (NIH) was used to quantify vGAT punctate staining and the ITCN nuclear count tool was used to quantify Hoechst positive nuclei. Each data point represents a region of interest (ROI). Total n per group is 4 sections, one per organoid and each belonging to independent derivations. Comparison between groups was performed with an unpaired t test with Welch’s correction (*p < 0.05)

Previous studies in mouse models reported changes in presynaptic release and region-dependent neural network activity dysfunction due to *CLN3* deficiency, especially affecting GABAergic and glutamatergic transmission, particularly in the amygdala, hippocampus, and cerebellar networks [13, 28]. Immunofluorescence stainings for synaptophysin (presynaptic marker) and postsynaptic density protein 95 (PSD95; postsynaptic marker) revealed a significant decrease in presynaptic and postsynaptic counts in CLN3^Q352X^ mutant organoids (Fig. 6a, b). These synaptic marker proteins have been shown to manifest changing patterns during the human frontal cortex development [24], but alterations in the levels of these proteins in the cerebral organoids model complement the gene expression data (Fig. 5) highlighting impairments in synapse formation. To gain further insights, we compared the metabolic profiles of CLN3^Q352X^ mutant cerebral organoids to the isogenic controls. Unsupervised clustering separated both genotypes, highlighting dysregulations in 66 different metabolites between the two, with most of the metabolites being less represented in the CLN3^Q352X^ mutant organoids (Fig. 6c). From these metabolites, a total of 31 were identifiable (Additional file 6: Table S3). Within these metabolites, we detected a significant decrease in some amino acids, such as tryptophan and lysine and interestingly also in neurotransmitters, like the γ-aminobutyric acid (GABA). Creatinine, a putative metabolite biomarker for several neurodegenerative diseases [40], was also significantly downregulated in the mutant organoids (Fig. 6d, e, Additional file 6: Table S4). To further demonstrate the downregulation of the GABA system, the vesicular GABA transporter vGAT was checked via immunostaining, revealing a significant decrease in the evaluated CLN3^Q352X^ mutant organoid sections (Fig. 6f, g). In summary, these findings reveal an impairment in synapse formation and neurotransmitter production in CLN3 mutant cerebral organoids.

## Discussion

The impact of CLN3 mutations on neurodevelopment is clearly understudied and especially studies using human in vitro models are missing. The advent of iPS cell technologies allows the development of such models, which are especially relevant in rare disease studies, where the paucity of available patient samples limits research development. In this context, the generation of isogenic cell lines by means of CRISPR/Cas9-mediated genome editing is of great importance, especially to isolate the effect of the disease-causing mutations from the patient-specific genetic background. Moreover, advances in three-dimensional organoid cultures foster the use of the human iPS cells to study neurodevelopment [1, 45]. Here, we generated a CLN3 mutant hiPS cell line by introducing the c.1054C → T pathologic variant in the *CLN3* gene and used organoid technology to obtain a neurodevelopmental in vitro model of CLN3 disease, which recapitulates key disease features and allows to study the impact of the mutation on brain development.

While characterizing the isogenic cell pair, we discovered that the introduced mutation promotes the exclusion of the constitutive mutated exon from the transcript. More importantly, we were able to confirm the existence of the alternative spliced variant in patient cells carrying the same mutation. This phenomenon was not previously reported for this mutation and is most likely occurring in other nonsense *CLN3* mutations located in exon boundaries, gaining specifically relevance when designing effective therapeutic strategies. In addition, the value of the here described isogenic pair was further demonstrated by showing distinct disease-specific phenotypes in different in vitro cultures. Firstly, in this study, hiPSC-derived endothelial cells recapitulate organellar pathology and present JNCL-type storage material. Secondly, hiPSC-derived cerebral organoids also displayed the disease hallmark, finger print material accumulation, interestingly found in combination with curvilinear bodies. Additionally, we show that storage pathology is co-occurring with alterations in the levels of lysosomal enzymes, such as TPP1 and CTSD, which have been described in various pathological conditions such as neurodegenerative lysosomal storage disorders, inflammation, cancer and aging [25, 39]. Taken together, these results validate the here described isogenic pair as a suitable human model for JNCL, which recapitulates key features of disease pathology both in two-dimensional non-neuronal and three-dimensional neural cell cultures.

Previous studies using cerebral organoids as model for rare diseases with known genetic risk factors, such as primary microcephaly [45] or Sandhoff disease [1] reported a markedly reduced or increased organoid size, respectively, with impaired neuronal differentiation in patient organoids. We observed that the *CLN3* mutant cells were considerably sensitive to the cerebral organoid differentiation, failing to form neuroepithelial buds after the start of differentiation and impairing their complete development. It is noteworthy that about half of the CLN3^Q352X^ organoids across the different organoid generations presented extremely severe development abnormalities, whereas the rest developed normally in terms of brain and cortical identity. Provided that the starting population for the organoid generation was a pool of gene edited hiPS cells, the maturation failure could be possibly due to different expression levels of the two mutant variants of the *CLN3* gene and dissimilar amounts of truncated protein. We hypothesize that compensatory mechanisms present within some of the edited cells may favour their further maturation. Therefore, we used the fully-developed organoids to unravel less severe disease signatures, caused by the presence of the CLN3^Q352X^ mutation, which may be present already during brain development and precede end-stage lysosomal storage defects. Transcriptomic profiling indicated that pathways involved in cell fate differentiation, proliferation and tissue and system developmental processes were altered in the CLN3^Q352X^ organoids compared to the controls. A closer look into subsets of genes appointed to specific downregulations affecting pathways related to corticogenesis and synapses. Although fully-developed organoids for both genotypes presented similar levels of cortical proteins, such as FOXG1 and SATB2, mRNA levels evaluated at the same time-point revealed downregulations for the corresponding genes in the CLN3^Q352X^ mutant organoids. We consider that, at the stage of analysis, the system is already showing alterations at the gene expression level, but is still able to compensate for these alterations by using the available transcripts to produce sufficient levels of the corresponding proteins. On the other hand, recent studies reported alterations in the synaptic compartment in CLN3 animal models revealing neurotransmission defects [28] and molecular regulators of synaptic stability [48]. Changes in proteins involved with synaptic function/stability were previously reported in mouse models of different NCL forms [37]. In the CLN3 mutant organoids, evaluation of pre- and post-synaptic proteins revealed a marked decrease in these proteins, suggesting potential alterations in synapse formation during brain development. To evaluate if synapse defects have an influence on neurotransmitter levels, we studied the metabolome of the mutant cerebral organoids, with special focus on metabolites that are implicated in neurotransmission. We were able to find imbalances particularly related to the GABA metabolism, but also in other relevant cerebral metabolites. This constitutes an interesting finding, since GABAergic interneurons are affected in JNCL disease pathogenesis in various animal models and human patients [50, 53]. The alterations discovered using the cerebral organoid model support the concept of a developmental component to JNCL pathology. We suggest that dysregulated gene expression in key pathways for development and synaptic alterations, particularly affecting GABAergic populations might precede the accumulation of pathologic storage material and the impairment of the lysosomal enzymatic profile, affecting the trajectory of cortical development in children with JNCL.

The advantage of using gene regulatory networks to link genes based on their interaction at the transcriptional level is that it allows detecting hub genes that could be candidate genes for modulation to revert the disease phenotype and bring the mutant network closer to the healthy one. As a proof of principle, we performed in silico network perturbations [84] to identify the most influential genes in the diseased network [36]. Single and multi-gene perturbations revealed several transcription factors, listed in Additional file 6: Table S5, that play an important role in maintaining the diseased phenotype network. Although the predicted genes are not necessarily responsible for disease onset and progression, they are predicted to be able to revert most of the diseased gene expression program upon perturbation and thus would be interesting candidates in future studies that could lead to a significant reversion of the pathologic phenotype upon modulation.

The acquisition of an isogenic pair in the context of a rare disease, such as JNCL, overcomes the primary limitation of access to patient material. Moreover, having hiPSC lines that can be differentiated into several cell types allows the study of the pathogeny and the mechanisms of the disease in the different tissues that may be affected. More importantly, the thorough analysis of a particular mutation tailors the study in a personalized manner, and raises the possibility of discovering specific drugs which can have a direct translational impact in patients carrying this particular mutation [10]. Additionally, the established three-dimensional cerebral organoid model for JNCL fosters human in vitro studies of early developmental consequences of this lysosomal storage disorder, where subtle abnormalities could be found preceding terminal cell death and neurodegeneration.

## Materials and methods

### Human iPSC culture

The characterized Gibco (Cat no. A13777) episomal human induced pluripotent stem cell line was chosen as control line to conduct the genome editing. Cells were normally cultured in Matrigel (Crorning, cat no. 354277) coated plates using Essential 8 medium (Thermo Fisher, cat no. A1517001) with 1% Penicillin/Streptomycin (Invitrogen, cat no. 15140122). Cell splitting procedures were performed using Accutase (Sigma, cat no. A6964) and plated in the same culturing media, but containing ROCK inhibitor Y-27632 (Merck Milipore, cat no. 688000) at 10uM for 24 h after dissociation.

### Construction of sgRNA vectors and donor plasmids

Introduction of a disease-causing point mutation into the healthy background A13777 hiPS cell line was performed as previously described [3, 35]. Briefly, sgRNAs targeting sequences to exon 13 of the *CLN3* gene were selected in silico using a previously published tool [20]. The selected Cas9 sequences with predicted high activity were then cloned into the pX330 vector (Addgene, 42,230) as described in Ran et al., 2013 [67]. Bi-allelic targeting of the CLN3 locus was achieved by assembling, using Gibson assembly (NEB, cat no. E2611L), the corresponding homology arms into two different donor scaffolds, carrying either EGFP (Addgene 100,603) or dTomato (Addgene 100,604) fluorescent reporters [3, 35]. Both donors contained the desired modification to achieve a homozygous insertion. The donor vectors were named A235-CLN3-donor-green-Q352X and A236-CLN3-donor-red-Q352X. All primers used are indicated in Additional file 6: Table S1 and sgRNA sequence resulting in successful editing was corresponding to CLN3-QtoO-B.

### Electroporation of parental cells and selection

Following normal dissociation, hiPSC were electroporated using the 4D-Nucleofector System (Lonza) and the P3 Primary Cell 4D-Nucleofector® X Kit (Lonza, cat no. V4XP-3024) following the manufacturer’s instructions and plated in media containing ROCK inhibitor Y27632 at 10uM for 24 h. Cells were cultured until small to medium size colonies were formed. Then, media was supplemented with 0.5 μg/mL puromycin (Sigma) until reaching enough confluence to perform FACS sorting of the whole population.

### Fluorescence activated cell sorting

Human iPSCs were enzymatically disaggregated and pellet was re-suspended in cold 1xPBS with 1% BSA (Carl Roth, cat no. 8076.3) and passed through a 20 μm pre-separation filter (Miltenyi, cat no. 130–101–812) to ensure having a single cell suspension. Cells were sorted using FACS ARIA III (BD Bioscience), using an 85 μm size nozzle and a 2.0 neural density filter. Single cell gating hierarchies on FSC and SSC width and height were applied (Additional file 1: Figure S1a) and purity mask was used. CS&T and drop delay calibrations were ensured prior to each sorting round.

### Cell transfections with transposase mRNA and genotyping

Removal of the selection module was performed by transfecting the cells with transposase PiggyBac excision-only mRNA [47] with the Stemfect RNA transfection kit (Stemgent, cat no. 00–0069) following manufacturer’s instructions and the protocol described in Arias-Fuenzalida et al., 2017 [3]. Double-negative population was again purified by FACS sorting of the whole population. Correct introduction of the mutation was then evaluated via Sanger sequencing of the flanking genomic region (Fig. 1c), using primers in Additional file 6: Table S1.

### Microarray karyotype

Genomic DNA from the parental (Control) and the edited (CLN3^Q352X^ mutant) cell lines was isolated using GenElute Blood genomic DNA Kit (Sigma, cat no. NA2020). Samples were sent and processed at the Life & Brain genomics facility from Bonn University, using Illumina iScan technology (Illumina).

### Evaluation of exon skipping in the CLN3^Q352X^ mutant line

Total RNA was isolated using the RNeasy Mini Kit (Qiagen, cat no. 74106) following the manufacturer’s instructions. An on-column DNase digestion step was performed with RNase-Free DNase Set (Qiagen, cat no. 79254). Complementary DNA was synthesized using High Capacity RNA-to-cDNA Kit (Invitrogen, cat no. 4387406). Reverse Transcription PCR reaction was performed using GoTaq G2 Hot Start Green Master Mix (Promega, cat no. M7423) using the primers listed in Additional file 6: Table S1.

### Derivation and culture of endothelial cells

Endothelial cells were differentiated from hiPS cells following a modified version of Prasain et al., 2014 protocol [64]. A density of 250.000 hiPS cells were seeded per well of a Matrigel-coated 6-well plate and cultured on Essential 8 with 1% Penicillin/Streptomycin plus 10uM ROCK inhibitor for 24 h (day minus three). Media was exchanged at day minus two for MEF conditioned media (R&D Systems, cat no. AR005) supplemented with 10 ng/mL bFGF (PreproTech, cat no. 100-18B). At day minus one, media was refreshed completely with the same media and the addition of 4X Matrigel. Media was changed at day zero to RPMI media (Thermo, cat no. 21875–034) with the addition of 1X B27 without vitamin A (Life Technologies, cat no. 12587–10) and 10 ng/mL Activin A (Life Technologies, cat no. PHC9564) to direct cells toward mesodermal lineage. From day one onwards, cells were cultured in RPMI with B27 media, with the addition of 10 ng/mL bFGF, 10 ng/mL BMP4 (PreproTech, cat no. 120–05) and 10 ng/mL VEGF165 (PreproTech, cat no. 100–20). Cells were sustained in differentiation media conditions for two additional weeks.

### Generation and culture of human cerebral organoids

Human whole brain organoids were derived from the isogenic pair hiPSCs following the Lancaster and Knoblich, 2014 protocol [44]. Cerebral organoids were maintained under differentiation conditions for 55 days after the embedding day (total 66 days).

### Electron microscopy

Cultured cells and organoids were fixed using a modified Karnovsky’s fixative (pH 7.4), containing 2% PFA and 2% glutaraldehyde in 0.1 M sodium cacodylate buffer for 3 h at RT on a shaker. After this time, fixative was replaced with fresh solution and organoids were incubated for 2 days at 4 °C on a shaker. Organoids were then rinsed several times with 0.1 M cacodylate buffer. Cultured cells were partially scraped and concentrated in a pellet then re-suspended in 2% low melting point agarose, the other part of the monolayer was flat embedded. All samples were post-fixed in 1% osmiumtetroxide, 1.5% potassiumferrocyanide in 0.1 M cacodylate buffer and stepwise dehydrated in ethanol, including in-bloc 0.5% uranyl acetate staining during 70% ethanol incubation. Samples were embedded in epon and sectioned ultrathin at 70 nm. Sections were collected on copper grids and stained with lead. Samples were analyzed on a Tecnai 12 electron microscope (Thermofisher Scientific, Eindhoven, The Netherlands) and representative areas were documented with a 2 K CCD-camera (Veleta, EMSIS, Münster, Germany). Three different blocks of each condition were sectioned and examined. Cultured endothelial cells were analysed as scraped cell sample, thus randomly orientated. Flat embedded cells were investigated in a polar manner from basolateral to apical side. Organoid samples were sectioned in three different orientations: peripheral, cross-sectioned through the centre and sectioned from the centre part of the organoids. Representative pictures are shown in the corresponding figures in the results section.

### Immunofluorescence in cells

Cell fixation was done using 4% PFA in PBS for 15 min at RT. Cells were washed then 3x with PBS for 5 min at RT and permeabilized using 0.05% Triton-X100 in PBS for 10 min at 4 °C. Blocking was performed for 1 h at RT with 10%FCS in PBS. Incubation with the corresponding primary antibodies at the required concentrations was done overnight at 4 °C in blocking buffer. For pluripotency stainings, SSEA4 (Abcam, cat no. ab16287, 1:50), Oct4 (Abcam, cat no. ab19857, 1:400), TRA-1-60 (Millipore, cat no. MAB4360, 1:50), TRA-1-81 (Millipore, cat no. MAB4381, 1:50), Nanog (Millipore, cat no. AB5731, 1:200) and hSox2 (R&D Systems, cat no. AF2018, 1:100) were used. Endothelial cells were characterized by the expression of CD31 (Dako, cat no. M082301, 1:100) and stained for SCMAS (Abcam, cat no. ab181243, 1:100), LAMP1 (Abcam, cat no. ab25630, 1:100), GM130 (BD Bioscience, cat no. 610822, 1:200) and Rab7 (Abcam, cat no. ab50533, 1:200) for subcellular studies. Incubation with secondary antibodies was done after 5 min 3x washing steps with PBS, for 2 h at RT in blocking buffer. All secondary antibodies (Invitrogen) were conjugated to Alexa Fluor fluorochromes and used in a 1:1000 dilution together with a nuclei counterstain by Hoechst 33342 (Life Technologies, cat no. H21492). Cells were washed 3x with PBS, and either mounted with Fluoromount-G mounting medium (Southern Biotech, cat no. 0100–01) or covered with PBS and imaged directly after.

### Immunofluorescence in organoid sections

Organoids were fixed with 4% PFA overnight on a shaker at 4 °C and washed 3x with PBS. Afterwards, they were embedded in 3% low-melting point agarose (Biozym, cat no. 840100) in PBS and incubated at RT until the agarose solidified. The agarose block was either covered with PBS and kept at 4 °C or sectioned immediately. If not indicated otherwise, 80 μm sections were cut using a vibratome (Leica VT1000s), set to a speed of 6.1 and a frequency of 9. Sections were permeabilized and blocked in one-step with PBS containing 0.1% sodium azide, 0.1% sodium citrate, 5% goat or donkey serum and 2% BSA, for 90 min at RT on a shaker. Incubation with the corresponding primary antibodies at the required concentration was performed in blocking buffer containing 0.1% Triton-X for 48 h at 4 °C. Synaptophysin (Abcam, cat no. ab8049, 1:50), PSD95 (Invitrogen, cat no. 51–6900, 1:300), vGAT (Sigma, cat no. HPA058859, 1:200), TUJ1 (Millipore, Cat no. AB9354) and MAP 2 (Abcam, cat no. ab32454, 1:100) antibodies were used to evaluate synaptic markers. GFAP (Millipore, cat.no. AB5541, 1:1000) and MAP 2 (Abcam, cat no. ab32454, 1:100) antibodies were used for astrocytosis assessment. CTIP2 (Abcam, cat no. ab18465, 1:100), SATB2 (Abcam, cat no. 34735, 1:100), TBR1 (Abcam, cat no. 31940, 1:300) and FOXG1 (Abcam, cat no. 18259, 1:1000) antibodies were used for brain organoid characterization. SOX2 (Abcam, cat no. ab97959, 1:200) and Nestin (BD Bioscience, cat no. 611659, 1:600) primary antibodies were used for neural stem cell evaluation. After 3x wash with PBS for 15 min at RT, secondary antibodies were added in a solution of PBS with 0.05% Tween-20. All secondary antibodies (Invitrogen) were conjugated to Alexa Fluor fluorochromes and used in a 1:1000 dilution together with a nuclei counterstain by Hoechst 33342 (Life Technologies, cat no. H21492). Incubation was done for 2 h at RT on a shaker and protected from light. Sections were washed 3x with 0.05% Tween-20 in PBS for 5 min at RT and 1x with water and then mounted with Fluoromount-G mounting medium (Southern Biotech, cat no. 0100–01) on a glass slide. Sections were dried overnight at RT and in the dark.

### Western blotting

Cerebral organoids were individually lysed in a Urea containing lysis buffer (7 M Urea, 2 M Thiourea, 2% CHAPS and 1 M DTT) containing 1X protease inhibitor cocktail (Sigma). Protein samples were resolved by denaturing SDS polyacrylamide gel electrophoresis (SDS-PAGE) in 15% polyacrylamide gels. Proteins were transferred from the gel to PVDF membranes in an iBlot2 device (Thermo). Membranes were incubated with SuperSignal West Pico Chemiluminescent Substrate (Thermo, cat no. #34580). Enhanced chemiluminiscent signal was detected in a STELLA imaging system. Evaluated antibodies were TPP1 (Abcam, cat no. ab96690, 1:1000), SCMAS (Abcam, cat no. ab181243, 1:1000), RIP1 (BD Bioscience, cat no. 610459, 1:1000), RIP3 (Abcam, cat no. ab152130, 1:1000), FOXG1 (Abcam, cat no. 18259, 1:500), SATB2 (Abcam, cat no. 34735, 1:250) and GAPDH (Abcam, cat no. 9485, 1:1000).

### Human Cathepsin D ELISA detection

Cathepsin D levels were evaluated using the Human Cathepsin D SimpleStep ELISA kit (Abcam, cat no. ab213470) following the manufacturer’s instructions. Briefly, cerebral organoids were individually disrupted in 200 μL of chilled 1X PTR cell extraction buffer and incubated for 20 min on ice, while homogenizing with either a pipette tip or needles. Samples and standards were loaded into the 96-well assay plate strips and incubated for 1 h at RT together with the antibody cocktail. Wells were washed 3x in 1x PT wash buffer and incubated with TMB substrate for 10 min. Reaction was the stopped and optical density at 405 nm measured in a Cytation 5 plate reader (BioTek) as an endpoint measurement. For normalization, sample protein concentration was quantified using Protein Quantification Assay (Macherey-Nagel, cat no. 740967.250).

### Terminal deoxynucleotidyl transferase biotin–dUTP nick end labeling (TUNEL) assay

General apoptosis within the organoids was detected using the In situ cell death detection kit, TMR red (Merck, cat no. 12156792910) following the manufacturer’s instructions, adapted to screen organoid sections. Briefly, organoid sections were permeabilized with 0.1% Triton-X in 0.1% sodium citrate for 8 min at 4 °C. After 3x wash with PBS, sections were incubated with the TUNEL reaction mixture for 1 h at 37 °C and in a humidified atmosphere and in the dark. Hoechst 33342 (Life Technologies, cat no. H21492, 1:1000) was added to the mix to visualize the nuclei. Positive controls were treated in a previous step with 5uL of Deoxyribonuclease I (DNase I) (Sigma-Aldrich, cat no. AMPD1-1KT, 1unit/uL) for 10 min at RT to induce DNA strand breaks. Negative controls were incubated with label solution only. Following 3x wash with PBS, sections were mounted with Fluoromount-G mounting medium (Southern Biotech, cat no. 0100–01) on a glass slide. Sections were dried overnight at RT and in the dark. Imaging was performed in Operetta High-content Imaging System (Perkin Elmer) in a range of 520-560 nm excitation and 570-620 nm emission.

### RNA-Seq

Total RNA was isolated from cerebral organoids using the RNeasy Mini Kit (Qiagen, cat no. 74106). Six replicates per condition, constituted by a pool of three organoids per replicate, were extracted. RNA quality was assessed in a Bioanalyzer 2100 (Agilent). In a second step, library preparation for sequencing was done with 1 μg of total RNA using the TruSeq mRNA Stranded Library Prep Kit (Illumina) according to manufacturer’s protocol. Pooled libraries were sequenced on NextSeq500 using manufacturer’s instructions.

### RNA-Seq data processing and analysis

Illumina Hi-Seq single-end reads were filtered by using BBDuk (trimq = 10 qtrim = r ktrim = r k = 23 mink = 11 hdist = 1 tpetbominlen = 40; http://jgi.doe.gov/data-and-tools/bb-tools/) to remove illumina adapters, PhiX library adapters, and to quality trim the reads. FastQC [2] was used to check the quality of the reads in order to assure that only high-quality reads are retained for subsequent analysis. Resulting reads were mapped to human GRCh37 genome by using tophat (version 2.1.1) [75] (library-type = fr-secondstrand) and Bowtie2 (version 2.3.2.0). Obtained alignment files were sorted by using samtools (version 1.6–5) [46] and the statistics of the alignment rate were obtained by using samtoolsflagstat. Cufflinks (version 2.2.1) [75] was used to quantify the transcripts and resulting expression values per gene were obtained in FPKM (fragments per kb per million reads). Differential expression analysis between the Control and CLN3^Q352X^ mutant samples was conducted by using the cuffdiff program from the cufflinks tool. Only significantly, differentially expressed genes with an absolute log2 fold change greater than 1 were considered for subsequent analysis.

### Gene regulatory network reconstruction

For the set of significantly differentially expressed genes (DEGs), the experimentally validated direct gene-gene interactions were retrieved from MetaCore (Clarivate Analytics). The interaction types belonging to categories “Transcription regulation” and “Binding” were kept in the prior knowledge network (PKN) from MetaCore. Differential network inference method proposed by [84] was used to prune the network edges (interactions) which are not compatible with the discretized gene expression program of the respective phenotype. Briefly, this method uses discretized differential gene expression data and infers two networks representing the mutant (disease) and cotnrol (healthy) phenotypes as steady states. Some of the interactions derived from MetaCore have an unspecified regulatory effect, as the exact mechanism of regulation is not known in those cases. The proposed algorithm also infers the regulatory effect (activation or inhibition) for such unspecified interactions based on the given gene expression pattern.

### Identification of network perturbation candidates

The differential network topology allowed us to identify common and phenotype-specific positive and negative elementary circuits, i.e. a network path which starts and ends at the same node with all the intermediate nodes being traversed only once. These circuits have been shown to play a significant role in maintaining network stability [27] and the existence of these circuits is considered to be a necessary condition for having a stable steady (network) state [74]. Considering the importance of these circuits, it has been shown that perturbation of genes in the positive circuits induces a phenotypic transition [19]. Furthermore, the differential network topology also aids in identifying the differential regulators of the genes, which are common to both phenotype-specific networks. Altogether, the differential regulators and genes in the elementary circuits constitute an optimal set of candidate genes for network perturbation as they are able to revert most of the gene expression program upon perturbation. Identification of network perturbation candidates was carried out by using the Java implementation proposed by Zickenrott and colleagues [84].

### In silico network simulation analysis for phenotype reversion

The Java implementation from Zickenrott and colleagues [84] was used to perform the network simulation analysis by perturbing multi-target combinations of up to four candidate genes identified in the previous step. The used algorithm gives a ranked list of single- and multi-gene(s) combinations (4 genes maximally) and their scores, which represent the number of genes whose expression is being reverted upon inducing the chosen perturbation in the diseased network. If a single- or multi-gene(s) perturbation combination obtains a high score, it is indicative of its ability to regulate the expression of a large number of downstream genes, hence playing a crucial role in the maintenance and stability of the phenotype under consideration.

### Gene and pathway enrichment analyses

MetaCore (Clarivate Analytics) and EnrichNet [23] were used to conduct the gene ontology (GO) and pathway enrichment analysis. The set of upregulated genes in the diseased network were used to identify the most over-represented biological processes and molecular functions associated with the genes in the network. Similarly, the most enriched biological pathways associated with genes that are upregulated in the pathologic network phenotype were described. The same kind of analysis was conducted to show downregulations in biological processes and pathways.

### Metabolomics

Metabolites from cerebral organoids were extracted and subsequently prepared for non-targeted gas chromatography-mass spectrometry (GC-MS) measurements, as previously described [34]. Five replicates per condition, constituted by a pool of three organoids per replicate, were extracted. Organoids were homogenized using a bead mill homogenizer system (Precellys24) and ceramic beads to produce a homogeneous suspension. Metabolites were then extracted with water/methanol/chloroform, yielding a three-phase system, where polar metabolites are enriched in the upper phase, non-polar metabolites in the lower phase and cell debris form a solid interphase. Polar phase was transferred into a sample vial and solvents were evaporated in a rotary vacuum evaporator at − 4 °C until dry and stored at − 80 °C until GC-MS analysis. Subsequent metabolic profiling is performed with gas chromatography coupled to mass spectrometry on an Agilent 7890B GC - Agilent 5977A MSD system. Post processing of the data was performed with the MetaboliteDetector software (http://md.tu-bs.de). Data was then manually curated, removing any possible contamination of external compounds due to sample preparation and the internal standards.

### Image acquisition

Brightfield and fluorescence representative images of the gene editing procedure, cell culture characterization and organoid images for area measurements were acquired using an inverted microscope (Zeiss Axio ObserverZ1). Synapse and autofluorescence analyses were performed on organoid sections imaged on a confocal (Zeiss LSM 710) laser-scanning microscope. Sections were usually imaged at 20-x and either tile scanning of the whole organoid sections were acquired or random regions of interest (ROI) within the sections were selected. Images were further processed with Zen Software (Zeiss) and quantifications were performed on ImageJ (NIH). The high content microscope OPERA QEHS spinning disk microscope (Perkin Elmer) was used for 2D endothelial cell imaging, using a 63-x water immersion objective. On the other hand, Operetta High-content Imaging System (Perkin Elmer) was used to acquire volumetric images (Z-stacking) of the entire organoid sections (Tile-scanning). The 20-x objective was used for this purpose. In both cases, volumetric images of plated cells and 3D images of cerebral organoids –e.g. TUNEL assay- were analyzed in Matlab (Version 2017b, The MathWorks Inc.), with in-house developed image analysis algorithms which automate the segmentation of nuclei and allow structure-specific feature extraction. The expression level of any marker was expressed as positive pixel of the marker, and commonly normalized by the pixel count of Hoechst.

### Statistical analysis and graphical representation

All the statistical analysis were performed in GraphPad Prism (Version 6.01). Significance asterisks represent *P* < 0.05 *, *P* < 0.01 **, *P* < 0.001 ***, *P* < 0.0001 ****; ns stands for not significant.

## Supplementary information

**Additional file 1: Figure S1.** Characterization of the *CLN3* isogenic pair.

**Additional file 2: Figure S2.** Ultrastructural evaluation of different organelles in endothelial cells.

**Additional file 3: Figure S3.** Characterization of hiPSC-derived cerebral organoids.

**Additional file 4: Figure S4.** Gene regulatory networks.

**Additional file 5: Figure S5.** Gene expression levels of relevant proteins.

**Additional file 6: Table S1.** Oligonucleotides used in this study. (*) This sgRNA was used for the editing. **Table S2.** Off-target analysis. **Table S3.** Identifiable metabolite changes detected by unsupervised ANOVA hierarchical clustering. **Table S4.** List of significant (*p* < 0.05) metabolic changes detected by Mann-Whitney test between genotypes. **Table S5.** Top 10 key candidate genes from single- and multi-gene network perturbation simulation analysis.

## Data Availability

All original and processed data used in this study are publicly available at this doi: 10.17881/lcsb.2019130913.01.
