# Supplementary figures and images for: Synapse alterations precede neuronal damage and storage pathology in a human cerebral organoid model of CLN3-juvenile neuronal ceroid lipofuscinosis
Source: Acta Neuropathol Commun. 2019 Dec 30;7:222. doi: 10.1186/s40478-019-0871-7 (PMC6937812; doi:10.1186/s40478-019-0871-7)

Figure S1

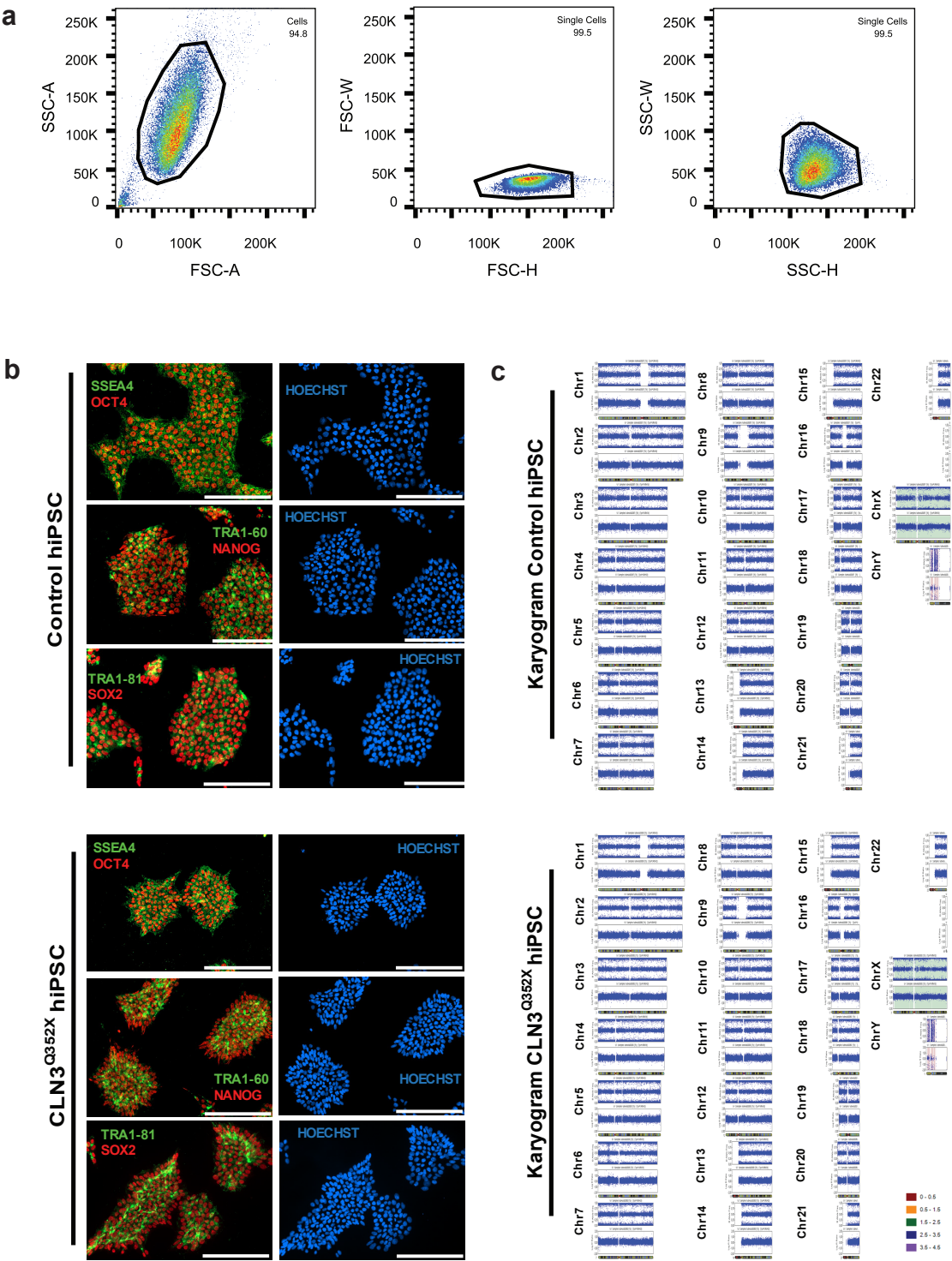

Supplement: Supplementary file 1 — Additional file 1: Figure S1. Characterization of the CLN3 isogenic pair. [file 40478_2019_871_MOESM1_ESM.pdf]

Figure S2

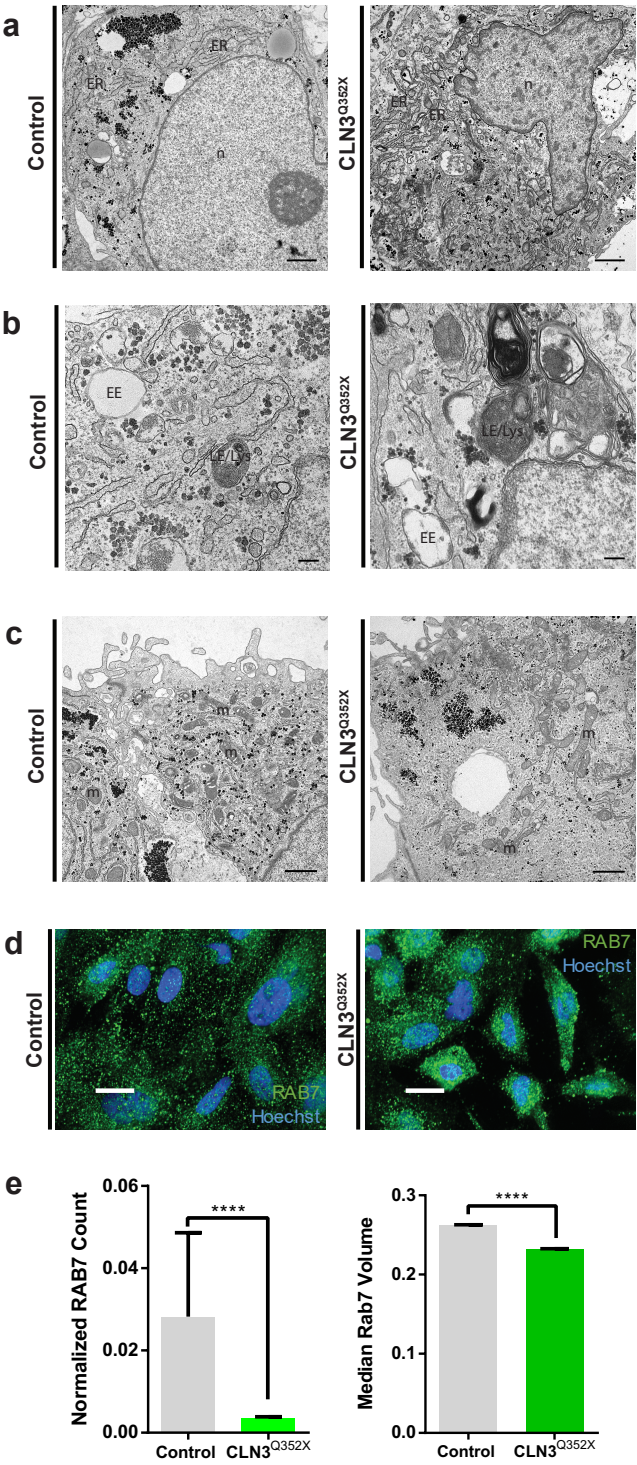

Supplement: Supplementary file 2 — Additional file 2: Figure S2. Ultrastructural evaluation of different organelles in endothelial cells. [file 40478_2019_871_MOESM2_ESM.pdf]

Figure S3

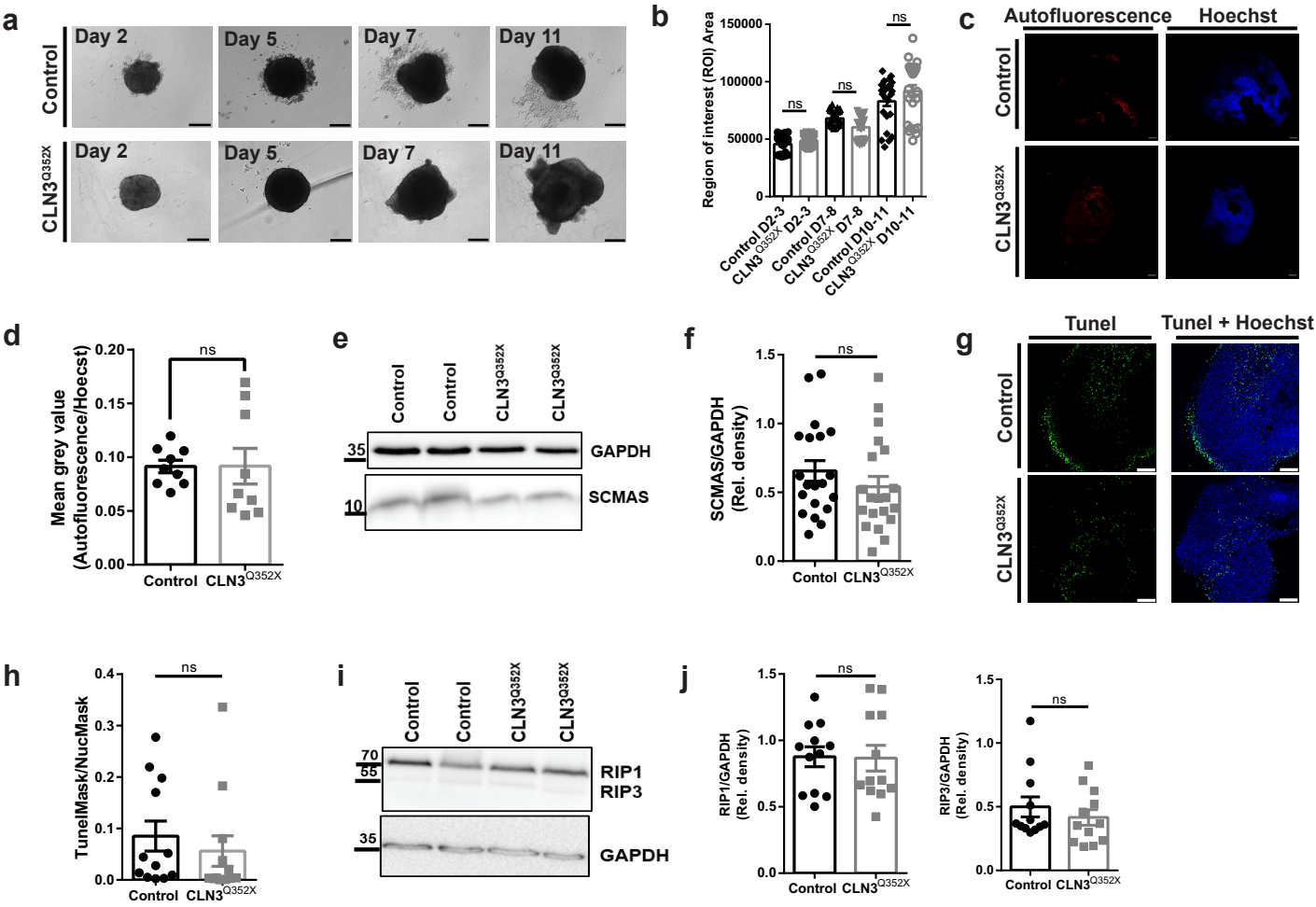

Supplement: Supplementary file 3 — Additional file 3: Figure S3. Characterization of hiPSC-derived cerebral organoids. [file 40478_2019_871_MOESM3_ESM.pdf]

Figure S4

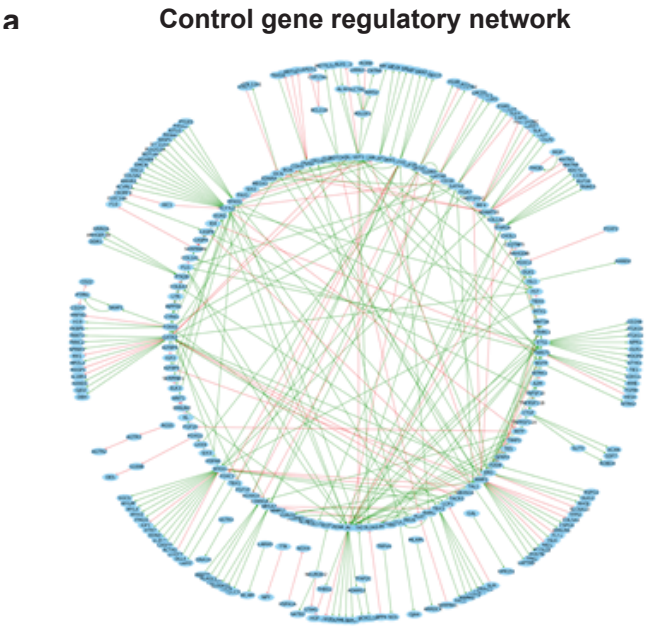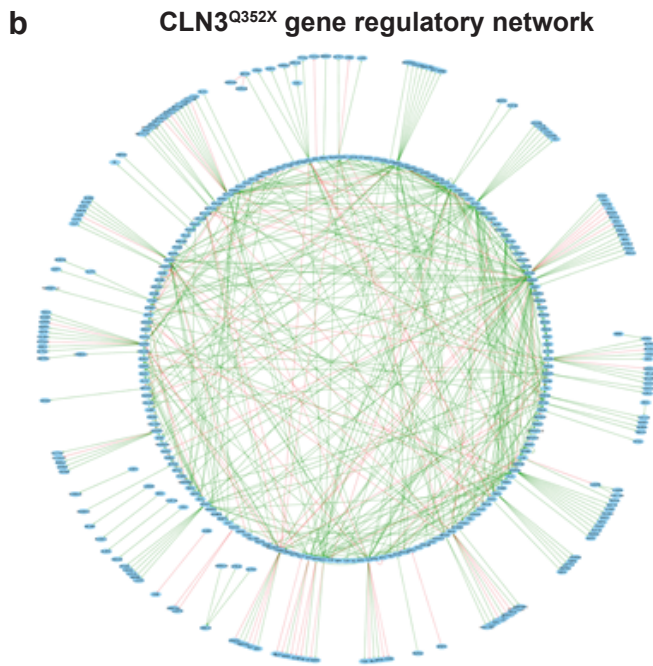

Supplement: Supplementary file 4 — Additional file 4: Figure S4. Gene regulatory networks. [file 40478_2019_871_MOESM4_ESM.pdf]

Figure S5

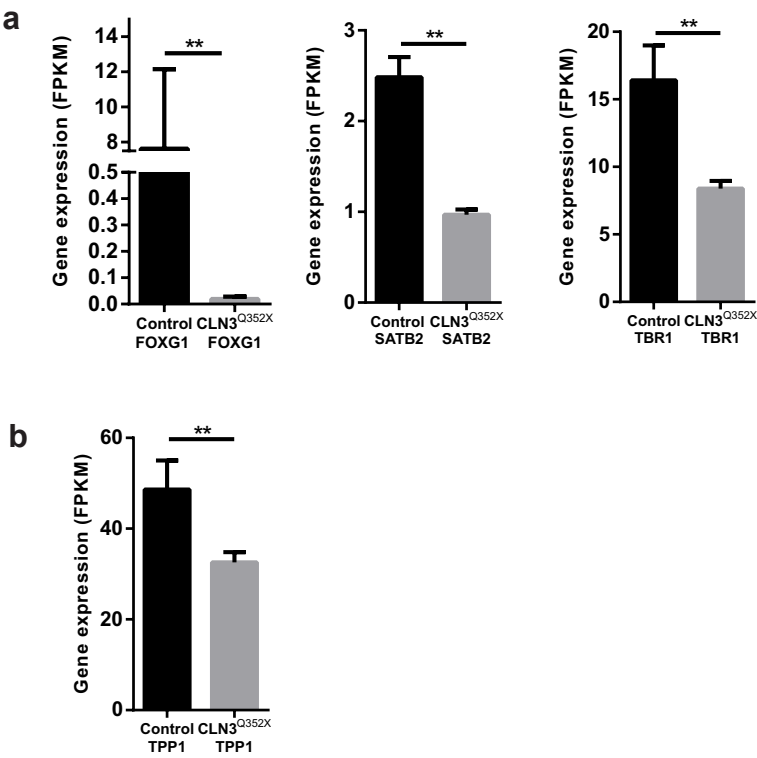

Supplement: Supplementary file 5 — Additional file 5: Figure S5. Gene expression levels of relevant proteins. [file 40478_2019_871_MOESM5_ESM.pdf]
