# Supplementary material for: Synapse alterations precede neuronal damage and storage pathology in a human cerebral organoid model of CLN3-juvenile neuronal ceroid lipofuscinosis
Source: Acta Neuropathol Commun. 2019 Dec 30;7:222. doi: 10.1186/s40478-019-0871-7 (PMC6937812; doi:10.1186/s40478-019-0871-7)
Supplement: Supplementary file 6 — Additional file 6: Table S1. Oligonucleotides used in this study. (*) This sgRNA was used for the editing. Table S2. Off-target analysis. Table S3. Identifiable metabolite changes detected by unsupervised ANOVA hierarchical clustering. Table S4. List of significant (p < 0.05) metabolic changes detected by Mann-Whitney test between genotypes. Table S5. Top 10 key candidate genes from single- and multi-gene network perturbation simulation analysis. [file 40478_2019_871_MOESM6_ESM.docx]

**Supplementary Tables**

**Table S1.** Oligonucleotides used in this study. (*****) This sgRNA was used for the editing.

| **Primer** | **Sequence (5’ to 3’)** | **Region (Purpose)** |
| --- | --- | --- |
| Q352oF1 | gtctcacctggttagcttttattttcatcatg | CLN3e10-13 (gDNA amplification) |
| Q352oF2 | cggtggggataagctgggatg | CLN3e10-13 (gDNA amplification) |
| Q352oR1 | tttgaaagcctgtctctttgcctg | CLN3e10-13 (gDNA amplification) |
| Q352oR2 | tactgtttgccaagggcagttg | CLN3e10-13 (gDNA amplification) |
| CLN3e10-13FmutP | ctctcgctcttctctgcgctgctgtcgcatccgtttcacc | CLN3e10-13 (PAM mutagenesis) |
| CLN310-13mutPR | agcgcagagaagagcgagaggcaaagacgccagcctggta | CLN3e10-13 (PAM mutagenesis) |
| CLN3-Q352oRHAF | atgattatctttctagggttaactcccaccttggctcccag | CLN3e10-13 (RHA assembly) |
| CLN3-Q352oRHAR | gcatacgcgtatactaggtttttgaaagcctgtctctttgcctg | CLN3e10-13 (RHA assembly) |
| CLN3-Q352oLHAF | aagcttggatcccctaggttgtctcacctggttagcttttattttatcatg | CLN3e10-13 (LHA assembly) |
| CLN3-Q352oLHAR | cagactatctttctagggttaagtgaggggcaggggtttggtacctacagcagggccagggcccaggtg | CLN3e10-13 (LHA assembly) |
| CLN3-QtoO-AF | caccgttacacttcccactgatagt | CLN3e10-13 (sgRNA cloning) |
| CLN3-QtoO-AR | aaacactatcagtgggaagtgtaac | CLN3e10-13 (sgRNA cloning) |
| CLN3-QtoO-BF | caccgtgaaacggatgcgacagcag | CLN3e10-13 (sgRNA cloning)***** |
| CLN3-QtoO-BR | aaacctgctgtcgcatccgtttcac | CLN3e10-13 (sgRNA cloning)***** |
| CLN3-QtoO-CF | caccgccctgcttcccactatcagt | CLN3e10-13 (sgRNA cloning) |
| CLN3-QtoO-CR | aaacactgatagtgggaagcagggc | CLN3e10-13 (sgRNA cloning) |
| CLN3-QtoO-DF | caccggacagcagcggagagaagag | CLN3e10-13 (sgRNA cloning) |
| CLN3-QtoO-DR | aaacctcttctctccgctgctgtcc | CLN3e10-13 (sgRNA cloning) |
| CLN3-QtoO-EF | caccgccactgatagtgggaagcag | CLN3e10-13 (sgRNA cloning) |
| CLN3-QtoO-ER | aaacctgcttcccactatcagtggc | CLN3e10-13 (sgRNA cloning) |
| ITR_R1 | agatgtcctaaatgcacagcg | ITR (Genotyping) |
| ITR_F1 | cgtcaattttacgcatgattatctttaac | ITR (Genotyping) |
| CLN3-CDSF | atgggaggctgtgcaggctc | CLN3e10-13 (Genotyping) |
| CLN3-CDSR | tcaggagagctggcagaggaag | CLN3e10-13 (Genotyping) |
| CLN3Q352XseqR | tcccactgatagtgggaagc | CLN3e10-13 (Sequencing) |
| CLN3ex11RNAFw | gggtctgctgtggtacattg | RT-PCR |
| CLN3ex15RNARv | caggagagctggcagaggaa | RT-PCR |

**Table S2.** Off-target analysis.

| **Chromosome** | **Mismatches** | **Sequence** | **PAM** | **Gene** | **Modification** |
| --- | --- | --- | --- | --- | --- |
| Chr16 | 0 | TGAAACGGATGCGACAGCAG | CGG | CLN3 | Yes (On-target) |
| Chr17 | 4 | TATTAGGGATGCGACAGCAG | CGG | CRHR1 | No |
| Chr1 | 3 | TGGAAGGGAAGCGACAGCAG | AGG | RP11-456P18.2 | No |
| ChrX | 4 | TGAGAGTCATGCGACAGCAG | TGG | DMD | No |
| Chr12 | 4 | TGACGGGGAGGCGACAGCAG | TGG | SH2B3 | No |
| Chr2 | 3 | TGATAGGGATGAGACAGCAG | TGG | C2orf47 | No |
| Chr11 | 4 | GGACAGGGATGAGACAGCAG | TGG | GRAMD1B | No |
| Chr2 | 4 | TCATAAGGATGAGACAGCAG | AGG | AC016712.3 | No |

| **Relative increase in CLN3^Q352X^** | **Relative decrease in CLN3^Q352X^** |
| --- | --- |
| Pyruvic acid | Glycerol |
| 3-Hydroxymethiylglutaric acid | Pantothenic acid |
| Malic acid | Glycerol-3-phosphate |
| *Myo-inositol* | Creatinine |
|  | Proline |
|  | Pentose_1 |
|  | Serine |
|  | γ-aminobutyric acid |
|  | Pentose_2 |
|  | 3-Hydroxybutiric acid |
|  | Butanoic acid |
|  | Fructose |
|  | N-Acetyl-L-aspartic acid |
|  | Glycine |
|  | Threonine |
|  | Methionine |
|  | Leucine |
|  | Phenylalanine |
|  | Isoleucine |
|  | Glutamic acid |
|  | Valine |
|  | Erythronic acid |
|  | Cysteine |
|  | Alanine |
|  | Mannose |
|  | Aspartic acid |

**Table S3.** Identifiable metabolite changes detected by unsupervised ANOVA hierarchical clustering.

**Table S4.** List of significant (p<0.05) metabolic changes detected by Mann-Whitney test between genotypes. Arrows show the trend of the change in the CLN3^Q352X^ cerebral organoids compared to the Control.

| **Metabolite** | **P value** |
| --- | --- |
| Pyruvic acid ↑ | 0.0079 |
| meso-Erythritol ↓ | 0.0079 |
| Glycerol_3TMS ↓ | 0.0079 |
| Pantothenic acid ↓ | 0.0079 |
| Glycerol-3-phosphate ↓ | 0.0079 |
| Lysine ↓ | 0.0079 |
| Creatinine ↓ | 0.0079 |
| Proline ↓ | 0.0079 |
| Tryptophan ↓ | 0.0079 |
| Pentose_1 ↓ | 0.0079 |
| Serine ↓ | 0.0079 |
| γ-aminobutyric acid ↓ | 0.0079 |
| Tyrosine ↓ | 0.0079 |
| Carbonic acid ↓ | 0.0079 |
| Pentose_2 ↓ | 0.0159 |
| 3-Hydroxybutyric acid ↓ | 0.0159 |
| Succinic acid ↑ | 0.0159 |
| Spermidine ↓ | 0.0317 |

**Table S5. Top 10 key candidate genes from single- and multi-gene network perturbation simulation analysis.** Genes are ranked based on their score. The score represents the number of genes whose discretized expression is reverted (shifted from the pathologic towards the healthy phenotype) upon *in silico* perturbation. The scores obtained for different candidate genes are a qualitative measure of their ability to revert the disease phenotype.

| **Single-gene perturbation** | | | **Multi-gene perturbation** | | |
| --- | --- | --- | --- | --- | --- |
| **Rank** | **Score** | **Gene** | **Rank** | **Score** | **Gene** |
| 1 | 82 | FOXA1 | 1 | 118 | TAL1, GATA3, ETS1, RUNX1 |
| 2 | 69 | TAL1 | 2 | 116 | FOXA1, MYOG, MMP2, GATA3 |
| 3 | 67 | LEF1 | 3 | 114 | FOXA1, MMP2, GATA3, ETS1 |
| 4 | 67 | GATA3 | 4 | 114 | FOXA1, GATA3, ETS1, RUNX1 |
| 5 | 66 | MMP2 | 5 | 113 | FOXA1, MYOG, GATA3, ETS1 |
| 6 | 65 | RUNX1 | 6 | 111 | FOXA1, MYOG, GATA3, RUNX1 |
| 7 | 65 | ETS1 | 7 | 111 | FOXA1, MMP2, GATA3, RUNX1 |
| 8 | 64 | GATA6 | 8 | 110 | MYOG, FOXA1, MMP2, RUNX1 |
| 9 | 63 | MYOG | 9 | 110 | MYOG, FOXA1, GATA3, GATA2 |
| 10 | 63 | IRF4 | 10 | 110 | FOXA1, MMP2, GATA3, GATA2 |
